# A Multivariate Evaluation of 25 Proximal and Distal Risk-Factors for Gambling-Related Harm

**DOI:** 10.3390/jcm8040509

**Published:** 2019-04-13

**Authors:** Matthew Browne, Nerilee Hing, Matthew Rockloff, Alex M. T. Russell, Nancy Greer, Fiona Nicoll, Garry Smith

**Affiliations:** 1 Experimental Gambling Research Laboratory, School of Health, Medical and Applied Sciences, CQUniversity, University Drive Bundaberg, Branyan, QLD 4670, Australia; n.hing@cqu.edu.au (N.H.); m.rockloff@cqu.edu.au (M.R.); a.m.russell@cqu.edu.au (A.M.T.R.); n.greer@cqu.edu.au (N.G.); 2 Department of Political Science, University of Alberta, Edmonton, AB T6G 2R3, Canada; f.nicoll@ualberta.ca; 3 Faculty of Extension, University of Alberta, Edmonton, AB T6G 2R3, Canada; garrysm@ualberta.ca

**Keywords:** risk factors, gambling-related harm, gambling problems, impulsivity, early experiences, safe gambling practices, fallacies

## Abstract

Individual differences in the risk of developing gambling-related harm play an important role in theoretical models and practical interventions. The present study attempted comprehensive measurement and evaluation of 25 known risk factors for gambling-related harm in order to determine which factors provided large and unique explanatory power. We surveyed 1650 regular gamblers from an online panel, screening in 1174 (466 male) who passed all checks of attention and response consistency. We evaluated each risk factor based on bivariate correlations with harms, then made separate multivariate evaluations of proximal (e.g., gambling motivations) and distal (e.g., religiosity) risk factors. Almost all bivariate correlations were significant, but most distal factors were not significant in multivariate models. Trait impulsivity was the most important risk factor by a large margin. Excessive consumption, less use of safe gambling practices, and more fallacies were key proximal risks of harm. Many well-known correlates of gambling harm (e.g., youth, lower educational attainment) do not show a direct role in the development of gambling harm when controlling for other factors. The results support theoretical models that emphasise early conditioning and biological vulnerability (manifested through impulsivity). Since maladaptive cognitive and behavioural schemas appear to be more important than motivations (e.g., escape, excitement, ego), interventions may benefit by targeting these proximal drivers of harm.

## 1. Introduction

There are many sources of risk for gambling-related disorders and harm. Some of the most important are related to accessibility and gambling type, with the availability of continuous forms of gambling, (such as electronic gaming machines) a demonstrated risk factor at both the individual and population levels [1]. At the level of the individual, a wide variety of factors: Demographic, environmental, personality, and cognitive; have been identified as correlates or risks for gambling-related disorders and harm. However, much less is known about the relative importance of these risk factors in predicting gambling harm. Furthermore, the lack of true multivariate studies; i.e., those that attempt comprehensive measurement of risk factors for simultaneous analysis; prevents an understanding of which factors are likely to be direct influences, rather than exist as mere correlates. For example, it is commonly found that young people are at greater risk of experiencing harm from gambling. However, the size of this effect, relative to—and controlling for—other risk factors, is more obscure. Even less clear is whether the effect is direct or not; that is, whether it is still observed when potential mediating factors, such as trait impulsivity, are included in the model. The present study aims to assess which individual-level factors identified in the literature represent unique, direct, and meaningfully large risks for experiencing gambling harm. 

### 1.1. Risk Factors for What? Gambling Problems and Harm

Research on maladaptive gambling at the individual level varies in the degree to which the focus is on behavioural dependence, i.e., addiction, or alternatively, the negative consequences that arise from the excessive behaviour, i.e., harms [1,2]. Gambling problems are generally treated as a complex of both aspects, as measured by the widely-used Problem Gambling Severity Index (PGSI) [3]. Nevertheless, some conceptual work, e.g., Reference [4] has treated gambling problems primarily in terms of negative consequences. This is particularly useful when evaluating proximal risk factors, such as the endorsement of gambling-related fallacies, since they may have conceptual overlap with cognitive and behavioural aspects of gambling dependence or pathology. There are specific measures of harmful consequences [5,6,7], which avoid this issue, and therefore facilitate evaluation of proximal risk factors independent of the harms that they produce. The current study therefore focuses on gambling harm as the key outcome of interest. 

### 1.2. Risk Factors for Gambling-Related Harm

Availability of access, product characteristics, regulatory measures, environmental, and socio-cultural context are established factors that contribute to the risk of a particular person experiencing gambling harm [8]. Availability of access, product characteristics, regulatory measures, environmental, and social factors may all contribute to the risk of a particular person experiencing gambling harm [9]. However, a focus at the individual level is useful for understanding why some people, rather than others, develop problems. Many studies have noted individual-level correlates or risk factors for gambling problems and gambling harm. However, in a critical review, Johansson et al. [10] found only a few factors were ‘well-established’, where the conclusions could be supported by three or more well-performed studies. These included age, gender, cognitive distortions (erroneous perceptions, illusion of control), early gambling experiences, and comorbid states (mental illness, drug abuse). Alternatively, a systematic review and meta-analysis of longitudinal studies of risk factors among youth, revealed 13 individual risk factors; although most effect sizes were small [11]. The strongest reported effects were for gender (male), poor academic performance, impulsivity, and childhood temperament. Smaller, but reliable effects were observed for a number of substance-use comorbidities. Population studies tend to find that; in addition to being male, being ‘marginalised’; i.e., young, unemployed, or a member of an ethnic minority, presents a risk factor for gambling problems [12]. In a large (*n* = 15,000) Australian prevalence survey, identified risk factors included: Being young, speaking a language other than English, not working, living in a group household, and non-social/entertainment motivations for gambling; which may include to escape negative mood states or to win money [13]. 

### 1.3. Risk Factors, Comorbidities and Correlates

For the purpose of the present investigation, we identify risk factors from other correlates of gambling related harm based on whether they have a plausible direct role in the subsequent experience of harm from gambling. Longitudinal studies are arguably the gold-standard for demonstrating causality in gambling [14,15]. However, these studies are not without disadvantages, most notably attrition and expense [14]. In the analysis of cross-sectional data, it is crucial to apply an etiological theoretical framework, such as that of Williams et al. [4], in order to yield informative results. Certain risk factors, including demographics and generic stable traits, can arguably be safely treated as exogenous with respect to gambling harm. That is, there is a clear directed causal pathway from these variables to harm, and bidirectional effects are implausible or negligible. For example, a strong theoretical argument, partly supported by evidence [16,17] can be made for the following causal chain: Youthful age → risk taking → gambling harm → psychological distress, although the final link is plausibly bi-directional, as gambling may also be used to cope with negative affect. Nevertheless, such theoretical reasoning would advise on variables that should not be included as risk factors in a cross-sectional analysis, because of their strong consequential nature. Similarly, comorbidities/correlates, such as substance use are dubious risk factors, because their observed correlation with gambling harm is most plausibly the result of common underlying risks (a.k.a. ‘3rd variables’).

#### The Biopsychosocial Framework

The biopsychosocial framework is an etiological model that acknowledges that biological factors and early experiences with gambling may contribute to—and interact with—psychological factors, such as impulsivity, to contribute to the acquisition of problematic gambling behaviours [18,19]. Gambling-specific attitudes, beliefs, and expectancies may then develop through repeated exposure and conditioning, in order to promote maintenance of maladaptive behaviours and outcomes. The framework organises a diverse set of risk factors into two basic categories: Biological and environmental factors that contribute primarily to the acquisition; and gambling-specific cognitions and behaviours, that contribute primarily to maintenance and exacerbation [4]. Additionally, there are recognised conditions and impacts that tend to co-occur with gambling problems [20]. The degree to which these comorbidities are a result of common underlying vulnerabilities, as opposed to direct causal links, remains unclear.

From a structural perspective, non-specific environmental, biological and psychological risk factors can be thought of as exogenous or distal variables. They are factors for which a unidirectional influence on gambling harm is plausible. In contrast, gambling-specific cognitions, behaviours and motivations are closely linked to gambling-related harm; developing partly as a consequence of distal risks, and in tandem with each other through a process of repeated exposure and conditioning. Thus, they are endogenous or proximal to the outcome, and bi-directional relationships are plausible. Furthermore, they often mediate the effects of non-specific distal risk factors. For example, gambling fallacies have been found to mediate the relationship between superstitious thinking and problem gambling [21]. These mediating roles can offer insight into the mechanisms by which distal factors can contribute to the risk for gambling-related harm. 

### 1.4. The Present Study

Guided by the biopsychosocial framework [4,18], the present study attempts to make a comprehensive measurement of both proximal and distal risks for gambling harm in order to identify the most important direct variables in each category. An exploratory evaluation of the relative importance of each of the risk factors represents a first step towards translating etiological models of risk into measurable and testable models. Given the current ambiguity as to which variables and links are most important, we stop short of specifying and testing structural models (e.g., mediation, path analysis, structural equation modelling). 

In sum, we present separate multivariate analyses outlined below:

- Distal risks → gambling harm
- Distal risks → proximal risks
- Proximal risks → gambling harm.

## 2. Material and Methods

A cross-sectional online survey was conducted, that included measures of 17 distal and 8 proximal risk factors for gambling-related harm (25 total). We calculated a required sample size of 1043, based on a multiple regression with a maximum of 17 predictors, in order to detect a partial R2 of 0.01, with an alpha error probability of 0.05. Inclusion criteria were: Residing in Alberta Canada (location of the funding body); aged 18 years+; and at least monthly gambling (in aggregate) on VLTs/slots, casino games, bingo, instant win tickets, race betting, sports betting, keno, e-sports, and fantasy sports.

### 2.1. Participants and Screening

An accredited panel provider recruited participants for an online survey in late 2017, and compensated them with points that can be exchanged for rewards. Inclusion criteria were: Residing in Alberta Canada; aged 18 years or over; and engaging in at least monthly gambling. A total of 2041 people commenced the survey, and 1650 completed. Given the data were from an online panel, four checks were included (*n*, % failures) in order to implement a stringent threshold for inclusion, whilst still meeting the required sample size (item text in italics):

1. Basic compliance: *Please select Disagree for this item*. (112, 6.8%)
2. Basic compliance: *Please select ‘Almost always’ for this item* (217, 13.2%)
3. Consistent responses (reverse formed): *I keep a household budget*, *I don’t keep a household budget* (Either/or, 313, 18.9%)
4. Providing a non-required response: *Which of the following activities have you spent the most money on in the last 12 months?* (49, 2.9%)

Criteria (4) corresponded to types of gambling activities. Given that all participants gambled at least monthly, participants who did not check at least one activity were providing an inconsistent response. For the 1650 people who completed the survey, compliance with each individual response check ranged from 81.1% to 97.1%. However, a total of 476 (29.0%) were excluded based on failing at least one check, leading to a final sample size of 1174. Of these, 28.7% (338) gambled approximately once a month, 26.4% (310) gambled 2–3 times a month, 23.8% (280) gambled about once a week, and 20.9% (246) gambled 2–3 times a week or more. Table 1 provides demographic and descriptive statistics for the final sample.

### 2.2. Measures

Our selection of risk factors was informed by recent reviews of the literature [4,8,9,10,20,22,23,24], but was necessarily limited to factors that could be measured by self-report. 

#### 2.2.1. Distal Risk Factors

In the interest of maintaining a manageable number of variables in the multivariate model, contrast coding of some distal categorical variables; that is, a reduction in the number of coded categories; was informed by an initial bivariate comparison with the primary outcome of gambling harm (described below). 

We measured several demographics, including age and gender. Marital status was coded using two separate contrasts: Married and single (base categories: De facto, separated, and widowed). Education and income ordered categories were treated as integer scores (see Table 1). Those working part-time, unemployed or pensioners were combined in a single binary contrast (base: Full-time work, home duties, full-time students, self-employed, other). Carer status was incorporated in two contrasts: Whether (a) primary carer for another adult; or (b) dependent on another adult for primary care. Our preliminary comparisons showed that workers in Services, Trades, and Manufacturing sectors displayed similarly greater levels of harms, and these were combined in a single contrast.

Certain social and environmental gambling risk factors were also included as distal factors. Two questions probed childhood gambling experiences: (a) How often did you gamble with parents or accompany them when they gambled: Both coded never (0), sometimes (1), often (2), very often (3), and (b) whether any adults in the household had a gambling problem: No (0), mild (1), severe (2). In the biopsychosocial framework [4], these are treated as proximal factors (‘rewarding early gambling experience and subsequent conditioning’). However, we treat early experience as a distal factor, because of its early (childhood) temporal sequence. Concurrent/recent exposure and conditioning is partially captured in a separate proximal measure, described in the section below. The number of current friends who gamble was coded from (0) None to (4) Nearly all of them. Distance from home to a gambling venue where the participant can play VLTs/slots was coded from (0) <1 km, to (6) more than 100 km. 

Other psychosocial risk factors were considered. Respondents rated the personal importance of spirituality/religion on a scale from ‘not at all important’ (0) to ‘extremely important’ (4), and whether they had received a mental disorder diagnosis from a professional: No (0), yes (1). The Brief Perceived Social Support Scale (BPSS) [25] included 6 items (e.g., ‘I receive a lot of understanding and security from others’) measured on a 5-point scale (‘does not apply at all’, to ‘exactly applicable’). The Barratt Impulsivity Scale-Brief (BIS-B) [26] involves 8 items measured on a 4-point scale: ‘Rarely/never’ (0) to ‘almost always/always’ (3) and includes items, such as ‘I plan tasks carefully’ (R). 

#### 2.2.2. Proximal Risk Factors

The factor of ‘erroneous cognitions or lack of knowledge about gambling’ in the biopsychosocial framework [4] was captured using the Gambling Fallacies Measure (GFM) [27]. This 10-item questionnaire examined cognitive errors observed in gambling (such as ‘a positive attitude or doing good deeds increases your likelihood of winning money when gambling’). Responses are either coded as 0 (incorrect) or 1 (correct), and higher total scores reflect greater resistance to gambling fallacies. 

We included a measure of ‘Safe Gambling Practices’ (SGP), which is a new (paper in review) measure of self-regulatory gambling behaviours. This protective factor is intended to be complementary to the GFM, capturing adaptive behaviours, rather than maladaptive cognitions. It probes the use of nine behaviours using a yes/no (1,0) format, each of which is associated with avoiding gambling-related harm. The SGP includes items, such as ‘If I’m not having fun gambling, I stop’ and ‘I have a dedicated budget to spend on gambling’, measured using a binary response format. It also includes (reverse-coded) risky behaviours, such as ‘I use gambling to make money / supplement my income’ and ‘I have used cash advances on my credit card to gamble’. To ease interpretation of tables and figures, the SGP total scale was reverse coded in order to conform to the orientation of other risk factors, with increasing scores indicating increased risk.

Gambling motivations were assessed using the Gambling Outcomes Expectancies Scale (GOES; Flack and Morris 2016): This 18-item scale asks responses on a six-point scale (1 = strongly disagree, 6 = strongly agree). Total scores are generated for each of five domains of gambling motivation (social, money, excitement, escape, ego enhancement), with higher scores indicating more endorsement of that motivation.

As mentioned in the preceding section, early exposure to gambling was captured as a distal risk factor. However, our view is that recent/concurrent behavioural conditioning; including frequency of play on problematic forms, gambling alone, and gambling online; was treated as a proximal risk factor for gambling-related harm. Due to the lack of a validated measure, we settled for an ad-hoc approach: Combining measures of gambling frequency on common forms most likely to produce conditioning, and two gambling modes indicative of conditioning: Gambling online (0 = no, 1 = yes), and gambling alone (0 = never, 3 = almost always). The forms incorporated were video lottery terminals (VLTs), Keno, bingo and casino games (0 = not at all, 6 = four or more times a week). Thus, the sum over forms also partially captured the number of different forms played, which is also indicative of conditioning.

#### 2.2.3. Outcomes

Our primary outcome measure was the Short Gambling Harms Screen (SGHS) [5]. The measure is moderate- to highly correlated with the PGSI, but deliberately measures only adverse consequences rather than behavioural addiction: i.e., thoughts and behaviours associated with disordered gambling. The SGHS yields scores 0–10, based on yes/no responses to ten harmful outcomes (e.g., ‘increased credit card debt’. The SGHS raw scores are approximately linearly related to decrements in general health and wellbeing [5].

The Problem Gambling Severity Index (PGSI) [3] was also included. The PGSI contains nine items with four response options: ‘Never’ (0), ‘sometimes’ (1), ‘most of the time’ (2), and ‘almost always’ (3). Scores are summed, and can be used to categorise respondents as: Non-problem gambler (0), low risk gambler (1–2), moderate risk gambler (3–7), or problem gambler (8–27). In the present study, we applied a log (PGSI + 1) transformation to stabilise the variance. 

### 2.3. Statistical Analysis

Spearman correlations were used to assess bivariate effects. Multivariate regression was used to evaluate the unique effects of each risk factor. Distal factors were regressed on proximal factors, and both distal and proximal risk factors were regressed on gambling-related harm (SGHS) in separate models. Our focus was on determining the relative effect size of each risk factor, whilst controlling for other variables. Determination of unique partial effect sizes in a multivariate context is, in-principle, somewhat ambiguous [28], leading us to apply several approaches. Using ordinary least squares (OLS) regression, we calculated eta-squared, delta-R squared, and standardised beta coefficients [29]. We complemented these classical measures with a newer approach, called the ‘elastic net’ (EN), which is based on a penalised (or regularised) regression framework. In brief, a penalty based on the magnitude of the non-zero beta coefficient vector is added to the normal regression error term; and the weight of the penalty term is optimised via cross-validation. In contrast to OLS, in which beta coefficients are entirely unconstrained, the EN tends to drive non-informative effects towards zero (‘shrinkage’). This leads to the estimated beta coefficients being ‘conservative’; i.e., biased towards parsimony and robustness. In OLS, very large fitted beta coefficients may be observed, in which their functional role is largely ‘balancing out’ the effect of other correlated predictors (a.k.a. collinearity). EN regression prevents this phenomenon, leading to a much more natural interpretation as capturing the unique simple effect of each predictor. However, EN models do not include standard errors. The EN was implemented using the *glmnet* package [30]. All analyses were conducted in R [31].

### 2.4. Ethics

The study procedures were carried out in accordance with the Declaration of Helsinki. The Institutional Ethics Committee of BLINDED and BLINDED approved the study (approval numbers Pro00071513, H17/07-140). All subjects were informed about the purpose of the research, were aware their data were anonymous, that they could withdraw at any time, and all provided informed consent.

## 3. Results

Table 2 summarises regressions and correlations of distal risk factors on gambling harms (SGHS). With the exception of gender, all variables showed significant bivariate relationships to harms, in the expected direction. A total of 22.9% of the variability in harm was accounted for by the OLS multivariate regression. EN and OLS betas, eta- and partial r-squared all showed a consistent pattern. Trait impulsivity was the most important risk factor for gambling harm by a large margin, followed by early gambling experiences, (lack of) social support, and religion (being important in one’s life). Other distal risk factors were not significant. Risk factors that had relatively larger correlations with harm, but were not significant in the multivariate model included: Being younger, those working part time/unemployed/pensioners, not being married, having less education, and lower income.

**Figure 1:**
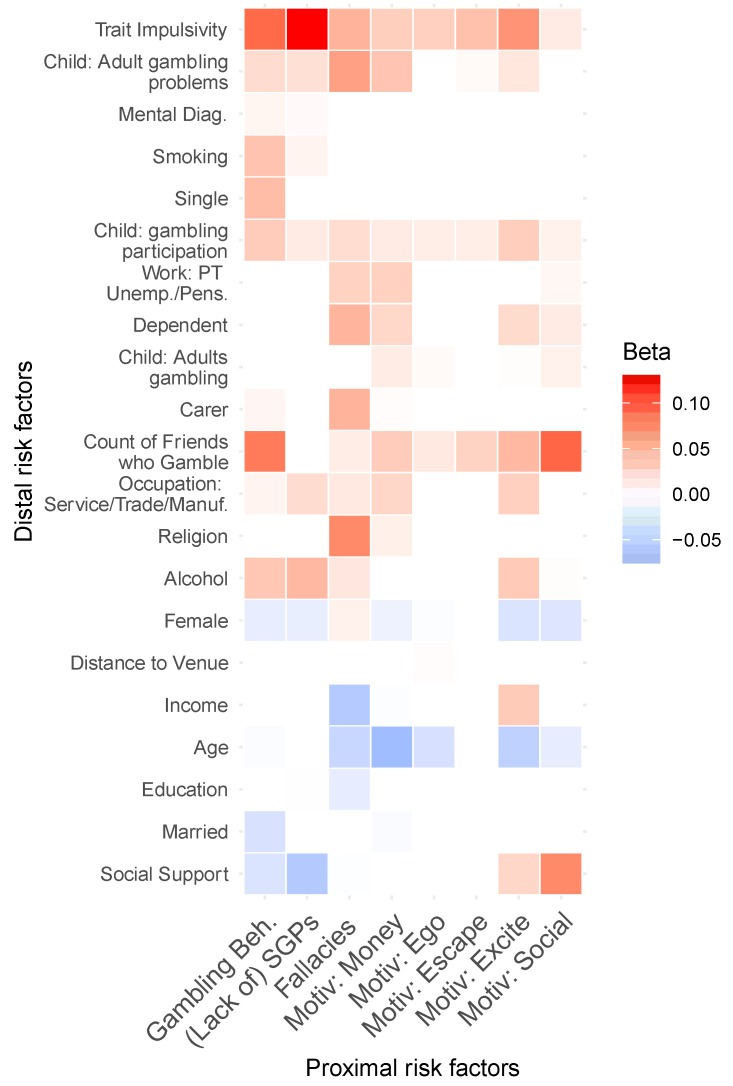
Elastic net regression coefficients on each of the proximal risk factors. Red indicates a positive relationship (greater risk) and blue indicates a negative relationship (less risk).

Figure 1 provides a graphical representation of the EN regression beta coefficients, of each of the distal risk factors on each of the proximal factors. Blank cells correspond to partial effects of zero. Rows are ordered with respect to the bivariate correlation of the risk factor with gambling-related harm. Trait impulsivity was a particularly important predictor with respect to (lack of) utilisation of SGPs; for which lack of social support was also an important predictor. Impulsivity, along with a count of the number of friends who gamble, were the strongest predictors of behaviourally-conditioned gambling. Fallacies were strongest amongst the religious, young people, and those with low income.

Table 3 summarises beta coefficients and effects size measures for an OLS model predicting gambling harm (SGHS) from proximal factors. EN coefficients and bivariate correlations with the SGHS are also shown. PGSI beta coefficients are also included for comparison purposes. A slightly higher R2 was observed for PGSI (44.1%) compared to SGHS (36.4%), which was expected given construct overlap between the predictors and the PGSI. Nevertheless, a similar pattern of effects is apparent across each of the multivariate indices. The use of SGPs, followed by gambling conditioning and fallacies, were the most important proximal determinants of gambling harm. The sum of the partial effects is much smaller (eta^2^: 17.66%, R2:11.19%) than the total variance explained (36.4%), indicating a high proportion of shared explanatory variance among the predictors. In this context, the EN coefficients are likely to provide a better indication of the relative importance of each of the predictors.

## 4. Discussion

The results help distinguish which risk factors are likely to play an important direct role in increasing risk for the experience of gambling-related harm.

### 4.1. Distal Risk Factors for Harm

By excluding proximal factors, and attempting comprehensive inclusion of distal risk factors, we are able to evaluate their unique influence on gambling related harm; whilst allowing that they may be partially mediated by more proximal variables. With the surprising exception of gender, all distal risk factors were found to be bivariate correlates of gambling-related harm; with direction conforming to prior findings. However, the multivariate regression showed a striking degree of parsimony: Trait impulsivity is the most important factor by a large margin, and only three other variables (having a family member with gambling problems as a child, social support, and religion) were significant at the *p* < 0.01 threshold. These variables—and especially trait impulsivity and childhood exposure to gambling problems—are therefore likely to be the key distal influences on risk for gambling harm as an adult. Therefore, our results suggest that remaining distal factors; including age, income, and education; are more properly considered correlates (or indirect risk factors) of harmful gambling, rather than having a direct causal link. An exploratory path analysis was beyond the scope of this study, due to a lack of theoretical clarity regarding model specification. Nevertheless, there are plausible mediating links between the correlates and the risk factors identified in this study, as well as between distal and proximal risk factors. For example, impulsivity—which is higher during youth [32]—may mediate a large proportion of the relationship between age and gambling-related harm. Gambling fallacies have been found to mediate the link between religiosity and gambling problems [33]; a result that was supported here.

#### Impulsivity

Our finding that impulsivity is the dominant distal risk factor is consistent with both Sharpe’s [18] biopsychosocial model and Blaszczynski and Nower’s [34] pathways model; both of which emphasise impulsivity as a key trait risk factor; itself partly determined by early experiences and biological influences, most notably dopaminergic dysfunction [35,36]. Although our focus was on harmful, rather than pathological gambling, this result runs counter to the reclassification of the disorder in the DSM-5 as an addiction, rather than an impulse control disorder [37]. We did not measure related constructs, such as sensation-seeking or self-control, nor more granular aspects of risk-taking, such as distinguishing between rash impulsivity and behavioural activation (itself comprising reward responsiveness, drive, fun-seeking) [38]. Distinguishing these traits may better capture risk for harmful or problematic gambling [39,40,41,42]. Given the importance of impulsivity found in the present analysis, and the role of dopaminergic functioning in influencing impulsive and reward-driven approach behaviour, further investigation on cortical and biological aspects of maladaptive gambling should be encouraged, particularly in relation to gambling formats designed to activate dopaminergic responses [43].

### 4.2. Proximal Risk Factors

The lack of a major direct role for the GOES in predicting harm appears reasonable. Motivations are understood in terms of perceptions of hedonic, tangible or social benefits from gambling [44]. As such, they are likely to contribute to frequency and type of gambling behaviours, as well as the likelihood of engaging in safe gambling practices. However, harm from gambling is understood as arising from excessive time and money actually spent on gambling—not from the psychological states or cognitions contributing to the desire to gamble. To some degree, the same could be said to be true for fallacies and SGPs. In theory, they should contribute to lower levels of excessive gambling, rather than directly causing lower levels of harm. The fact that they do explain unique variance in the degree of harm potentially points to the inadequacy of our behavioural measure to fully capture the concept of excessive gambling.

### 4.3. Measuring Behaviourally Conditioned or Excessive Gambling

In lieu of a better alternative, the present study employed an ad-hoc behavioural measure, including frequency of gambling on certain forms, gambling alone, and gambling online. Unfortunately, to our knowledge, a precise measure of conditioned or excessive gambling—expressed purely in behavioural terms—does not exist. It is in-principle difficult to operationalise this construct, because the ‘non-excessive’ limits for time and money spent may vary greatly from person to person [45] and in practice, may only be most apparent to the gambler via the effects (in terms of harms) after this theoretical limit have been exceeded. In practical terms, it is difficult to assess discretionary time and income, as well as time and money spent on gambling over an extended period [46]. A viable alternative might be to assess a variety of indicators of excessive gambling that are not also markers of pathology or harm. These might include items, such as ‘I sometimes spend more on gambling than I intended to’, ‘Sometimes when I finish gambling, I’m surprised by how much time has passed’, ‘I probably spend more on gambling than most people in my situation’. The frequency of play beyond recommended limits, as well as gambling alone or gambling on multiple or more problematic forms, could also form part of this measure. On theoretical grounds, an ideal measure of this kind would fully mediate the relationship between SGPs, fallacies, pathology/dependence, and other risk factors on gambling-related harm.

### 4.4. Limitations

The present study was constrained by the well-known limits of the cross-sectional data analysis. This entails that causality from associative links must be inferred on theoretical grounds, rather than demonstrated through experimental manipulation or examined as longitudinal effects. The degree of ambiguity depends on the variables concerned. For example, the inference regarding a one-way causal link between religiosity and gambling harm (possibly fully mediated by fallacies) is relatively strong. However, in the case of social support, it is likely that some portion of this relationship may be bi-directional: Gambling problems leading to relationship deterioration and a decrease in social support. 

The second major limitation of the study was the lack of validated measures for certain key constructs. The most notable examples were: (a) Childhood exposure to gambling and family gambling problems, and (b) increased / excessive gambling, or gambling consumption indicating behavioural conditioning. The measurement imprecision reflects both the broad-spectrum and inclusive approach taken, as well as the lack of suitable measures of certain constructs. Given the importance of trait impulsivity and early exposure, more detailed measurement of these constructs is clearly warranted. At the time of survey design, there was no ‘pure’ measure of addiction symptomatology or behavioural dependence available; i.e., a measure that did not also include substantial probes of gambling-related harm (as is the case for the PGSI). Ideally, this distinct construct of dependence/addiction should be included as a key proximal risk factor for excessive gambling and harm. Finally, the role of religiosity warrants further investigation, using more specific measures of magical thinking, spirituality or belief in the paranormal—which seem highly likely to mediate the observed relationship with fallacies. As in the case of trait impulsivity, more detailed measures of these constructs do exist, and should be employed in future studies.

## 5. Conclusions

**Figure 2:**
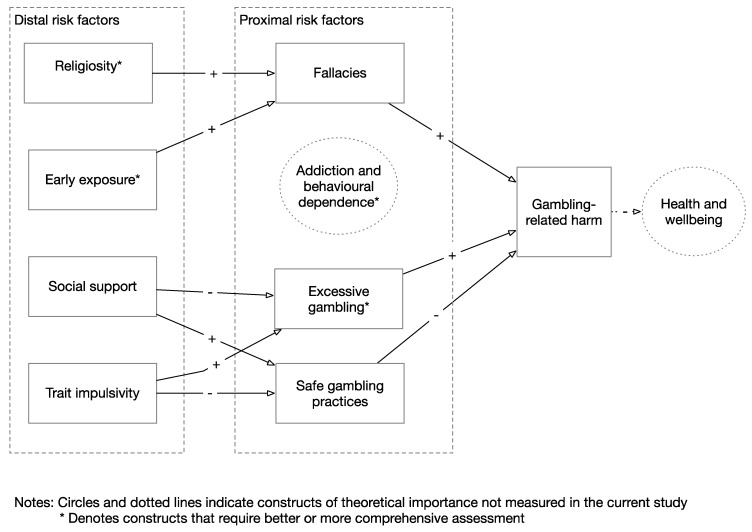
Conceptual diagram summarising relationships between risk factors identified in this study.

This was an exploratory study, intended to identify the key distal and proximal risk factors for gambling-related harm. Combined with mapping from distal to proximal risk factors, the results provide some clear directions for future measurement and specification of a more formal structural risk model. Figure 2 summarises the key findings, and includes only variables that appear to have a dominant causal role in the development of gambling-related harm. Also included for reference are two unmeasured variables: Addiction/behavioural dependence, and health and wellbeing; that we suggest should be included in future studies for a full theoretical understanding of risk for gambling-related harm. 

Most correlates of gambling harm or problems reported in the literature are not significant when considered in a multivariate context. The contribution of this paper is to focus our attention on those risk factors that provide large and unique explanatory power. Trait impulsivity, and early (childhood) exposure to gambling are key distal risk factors for gambling harm. Gambling fallacies, the (lack of) use of safe gambling practices, and excessive gambling consumption are the dominant proximal determinants of harm. Future research on proximal risk factors should include specific and validated measures of addiction/behavioural dependence, as well as excessive gambling, i.e., a profile of excessive gambling consumption indicative of conditioning. Future work on distal risk factors should focus on more comprehensive measurement of facets of trait impulsivity and early exposure to gambling. Further testing of the interaction between trait impulsivity and early exposure and certain types of gambling platforms and environments [47] may have significant implications for public policy and inform successful harm reduction strategies.

## Figures and Tables

**Table 1:** Demographic characteristics of the sample.

Variable	*(n = 1174) n (%)*
*Age*	Mean = 45.36 years (*SD =* 15.32)
*Gender*	
Male	466 (39.7)
Female	705 (60.1)
Other	3 (0.3)
*Residence*	
Calgary	393 (33.5)
Edmonton	366 (31.2)
Regional town	169 (14.4)
Small town	174 (14.8)
Rural or remote location	72 (6.1)
*Language spoken at home*	
English	1142 (97.3)
French	4 (0.3)
Other	28 (2.4)
*Indigenous status*	
Non-Aboriginal	1100 (93.7)
First Nation	32 (2.7)
Métis	42 (3.6)
Inuk (Inuit)	0 (0.0)
*Marital status*	
Single/never married	302 (25.7)
Living with partner/defacto	164 (14.0)
Married	538 (45.8)
Divorced or separated	131 (11.2)
Widowed	39 (3.3)
*Country of birth*	
Canada	1051 (89.5)
Other	123 (10.5)
*Living arrangements*	
Live alone	233 (19.8)
Couple (no dependents)	379 (32.3)
Couple with at least one dependent child	237 (20.2)
Couple living with independent child(ren)	85 (7.2)
Single parent living with at least one dependent child	59 (5.0)
Single parent living with independent child(ren)	31 (2.6)
Share house with other adults	74 (6.3)
Live with parents	60 (5.1)
Other	16 (1.4)
*Highest level of education*	
Grade 8 or less	3 (0.3)
Some high school	76 (6.5)
High school diploma or equivalent	287 (24.4)
Registered Apprenticeship or other trades certificate or diploma	113 (9.6)
College, CEGEP or other non-university certificate or diploma	325 (27.7)
University certificate or diploma below bachelor’s level	75 (6.4)
Bachelor’s degree	235 (20.0)
Postgraduate degree above bachelor’s level	60 (5.1)
*Work status*	
Work full-time	512 (43.6)
Work part-time or casual	165 (14.1)
Self-employed	89 (7.6)
Unemployed and looking for work	83 (7.1)
Full-time student	23 (2.0)
Full-time home duties	47 (4.0)
Retired	183 (15.6)
Sick or disability pension	58 (4.9)
Other	14 (1.2)
Occupation *	
Management	94 (8.0)
Business, finance and administration	92 (7.8)
Natural and applied sciences and related occupations	16 (1.4)
Health	89 (7.6)
Education, law and social, community and government services	80 (6.8)
Art, culture, recreation and sport	18 (1.5)
Sales and service	160 (13.6)
Trades, transport and equipment operators and related occupations	83 (7.1)
Natural resources, agriculture and related production occupations	18 (1.5)
Manufacturing and utilities	27 (2.3)
*Household income*	
$0 to $19,999	71 (6.1)
$20,000 to 39,999	170 (14.5)
$40,000 to $59,999	187 (15.9)
$60,000 to $79,999	174 (14.8)
$80,000 to $99,999	152 (12.9)
$100,000 to $119,999	102 (8.7)
$120,000 to $139,999	91 (7.7)
$140,000 to $169,999	70 (6.0)
$170,000 or more	69 (5.9)
Don’t know or refuse to answer	88 (7.5)
*Problem Gambling Severity Index (PGSI)*	
Non-problem	604 (51.4)
Low risk	276 (23.5)
Moderate risk	185 (15.8)
Problem	109 (9.3)
*Short gambling harms scale (SGHS)*	
0	682 (58.1)
1	138 (11.8)
2	86 (7.3)
3	76 (6.5)
4	42 (3.6)
5+	150 (12.7)

*Notes:* Count and percentages given unless otherwise specified. * Occupation was only asked for respondents who indicated they worked full-time, part-time, or casual, therefore *n* = 677 for this question. PGSI, Problem Gambling Severity Index; SD, standard deviation

**Table 2:** Bivariate correlations, ordinary least squares (OLS) and ‘elastic net’ EN multivariate regressions of distal risk factors on gambling-related harm.

	Multivariate Regression				Cor. (Spear.) ^+^
	Elastic Net	OLS			
	*B*	*B* (SE)	eta sq.	delta R sq.	
Trait Impulsivity	0.229	0.255 **(0.028)	6.54%	5.40%	0.315
Child: Family gambling problems	0.172	0.190 **(0.028)	3.90%	3.12%	0.277
Religiosity	0.080	0.091 **(0.027)	0.99%	0.77%	0.089
Social Support	−0.083	−0.089 **(0.027)	0.90%	0.70%	−0.153
Dependent	0.062	0.068 *(0.027)	0.54%	0.42%	0.082
Mental Diag.	0.060	0.060 *(0.028)	0.39%	0.30%	0.169
Single	0.059	0.066 *(0.033)	0.35%	0.27%	0.179
Female	−0.048	−0.057 *(0.028)	0.35%	0.27%	−0.008
Count of Friends who Gamble	0.045	0.049 (0.026)	0.30%	0.23%	0.099
Carer	0.045	0.045 (0.027)	0.25%	0.19%	0.118
Child: Gambling participation	0.043	0.040 (0.027)	0.18%	0.14%	0.146
Distance to Venue	−0.032	−0.035 (0.026)	0.15%	0.12%	-0.057
Occupation: Service/Trade/Manuf.	0.032	0.033 (0.027)	0.13%	0.10%	0.094
Work: PT Unemp./Pens.	0.035	0.034 (0.028)	0.13%	0.10%	0.131
Education	−0.035	−0.033 (0.028)	0.12%	0.09%	−0.129
Age	−0.028	−0.026 (0.031)	0.06%	0.05%	−0.141
Married	−0.021	−0.016 (0.033)	0.02%	0.02%	−0.129
Income	−0.014	−0.011 (0.030)	0.01%	0.01%	−0.123
SUM			15.31%	12.30%	
Observations		1174			
R2		22.90%			
Adjusted R2		21.71%			
Residual Std. Error		0.884 (df = 1156)			
*F*		19.07 **(df = 17; 1156)			

Note: * *p* < 0.05; ** *p* < 0.01, ^+^ All correlations significant at 0.01 threshold, OLS, except gender. ordinary least squares; EN, ‘elastic net’; PT Unemp., Part-time or unemployed; Pens., Pensioner; *B*, Standardised beta coefficient; SE, Standard error; eta sq., Eta Squared; delta R sq., Change in R-squared on deletion; Cor. (Spear.), Spearman non-parametric correlation.

**Table 3:** Bivariate correlations, OLS and EN multivariate regressions of proximal risk factors on gambling-related harm, and OLS multivariate regression of proximal risk factors on PGSI.

	log(PGSI + 1)	SGHS				
	OLS	EN	Cor. (Spear.)	*OLS*		
	*B* (SE)	*B*		*B* (SE)	eta sq.	delta R sq.
Gambling beh.	0.288 **(0.025)	0.241	0.43 **	0.244 **(0.026)	4.59%	2.81%
Safe Gambling Practices	0.353 **(0.024)	0.351	0.36 **	0.359 **(0.025)	10.46%	6.84%
Fallacies (GFM)	0.120 **(0.024)	0.136	0.26 **	0.140 **(0.025)	1.35%	0.80%
Motivations (GOES)						
Money	0.072 *(0.026)	0.072	0.28 **	0.073 **(0.028)	0.26%	0.15%
Ego	−0.012 (0.031)	−0.029	0.22 **	−0.037 (0.032)	0.68%	0.41%
Escape	0.066 **(0.027)	0.042	0.23 **	0.043 (0.031)	0.05%	0.03%
Excitement	0.116 **(0.030)	0.092	0.30 **	0.096 **(0.032)	0.21%	0.12%
Social	−0.021 (0.022)	−0.025	0.12 **	−0.027 (0.028)	0.06%	0.03%
Obs.	1174			1174		
SUM					17.66%	11.19%
R^2^	44.1%			36.4%		
Adj. R^2^	43.7%			36.0%		
Resid. SE	3.123			2.019		
F Statistic (df = 8; 1165)	98.843 **			83.357 **		

Note: * *p* < 0.05; ** *p* < 0.01; GFM, Gambling Fallacies Measure; GOES, Gambling Outcomes Expectancies Scale.
